# Cap-Independent Translation in Hematological Malignancies

**DOI:** 10.3389/fonc.2015.00293

**Published:** 2015-12-21

**Authors:** Emilie Horvilleur, Lindsay A. Wilson, Amandine Bastide, David Piñeiro, Tuija A. A. Pöyry, Anne E. Willis

**Affiliations:** ^1^Medical Research Council Toxicology Unit, Leicester, UK

**Keywords:** leukemia, lymphoma, myeloma, IRES, cap-independent, translation

## Abstract

Hematological malignancies are a heterogeneous group of diseases deriving from blood cells progenitors. Although many genes involved in blood cancers contain internal ribosome entry sites (IRESes), there has been only few studies focusing on the role of cap-independent translation in leukemia and lymphomas. Expression of IRES trans-acting factors can also be altered, and interestingly, BCL-ABL1 fusion protein expressed from “Philadelphia” chromosome, found in some types of leukemia, regulates several of them. A mechanism involving c-Myc IRES and cap-independent translation and leading to resistance to chemotherapy in multiple myeloma emphasize the contribution of cap-independent translation in blood cancers and the need for more work to be done to clarify the roles of known IRESes in pathology and response to chemotherapeutics.

## Introduction

Hematopoietic malignancies, here referred to as blood cancers, are the fourth most diagnosed cancer type in the world. In 2012, they had an estimated incidence of almost one million per year (6.5% of all cancers diagnosed) and an estimated mortality of over half a million, thus being responsible for ~6.9% of all cancer deaths (1). Hematopoietic malignancies can be classed as three broad groups: lymphomas which form tumors in the lymph nodes; leukemia cells that accumulate in the blood; and myelomas which are cancers of plasmocytes. Blood cancers result from genetic alterations in the hematopoietic lineage, leading to uncontrolled proliferation and/or resistance to apoptosis.

Hematopoiesis is a complex and tightly regulated process. In response to various signals, hematopoietic stem cells mature into either lymphoid or myeloid progenitors. Myeloid progenitors ultimately differentiate into erythrocytes, platelets, monocytes, and granulocytes (2). The lymphoid progenitor undergoes several subsequent maturation steps in the bone marrow and secondary lymphoid organs to produce B-cells, T-cells, and natural killer cells (NK). Malignancies can derive from any of these lineages and from cells at various differentiation stages; hence, the types of blood cancers are completely different diseases. Most hematopoietic malignancies occur in the elderly, but Hodgkin lymphoma (HL) affects young adults, and leukemias are the most common pediatric cancers. While some of these malignancies are very aggressive like acute lymphoblastic leukemia (ALL), acute myeloid leukemia (AML), and diffuse large B-cell lymphoma (DLBCL), others such as follicular lymphoma (FL), chronic myeloid leukemia (CML), or chronic lymphocytic leukemia (CLL) are relatively indolent and take several years to develop. They also have very different prognosis, e.g., 80% of HL can be cured while CLL remains incurable [for reviews, see Ref. (3–6)].

Despite the heterogeneity, many blood cancers share a common oncogenic mechanism. BCR and TCR genes undergo somatic recombinations to generate a broad range of receptors that would recognize various antigens. Off-target effects of these mechanisms can translocate proto-oncogenes to the highly transcribed BCR locus or lead to the expression of fusion proteins (7). While other mutations are found, the translocations are specific for each type of blood cancer and used for diagnosis and prognosis.

Differentiation and proliferation of blood cells is tightly controlled by an array of different extracellular signaling molecules, growth factors, and cytokines delivered by osteoblasts, osteoclasts, endothelial cells, stromal cells, mesenchymal progenitor cells, and adipocytes that populate the microenvironment of maturing hematopoietic cells: these components, together with the extracellular matrix, constitute the hematopoietic niche (8). While all hematopoietic malignancies interact with the hematopoietic niche, diseases in which no specific translocation is found (CLL and HL) are particularly dependent on their microenvironment and associated signaling pathways.

Various mechanisms deregulate cap-dependent translation in blood cancer, with the mTOR pathway being probably the most studied. mTOR is usually activated in aggressive diseases (ALL, AML, and DLBCL) resulting in an upregulation of translation. mTOR inhibitors, which should curtail translation, have been trialed in lymphomas, AML and CLL with mixed results (9). Protein synthesis can also be affected by deregulation of translation machinery components [e.g., eIF4E in leukemia (10) or eIF4B in DLBCL (11)], causing both global and gene specific effects. Finer gene-specific regulation of translation can be achieved with microRNAs, which can have either tumor-suppressor or pro-oncogenic effects. For example, miR146a expression is lost in lymphoma (12) and deletion of miR15/16 locus is common in CLL (13), while the miR17-92 cluster is a target of c-Myc and mediates its oncogenic effects [for review, see Ref. (14)].

When cap-dependent translation pathways have been downregulated, for example, through a change in microenvironment or presence of chemotherapeutics, cancer cells may still drive selective oncogene translation via “cap-independent” mechanisms. In such cases, an internal ribosome entry site (IRES) in the 5′ UTR of an mRNA interacts with a selection of canonical initiation factors and specific IRES trans-acting factors (ITAFs) to recruit ribosomes and translate protein independently of the 5′ cap. ITAFs are RNA-binding proteins that can either activate or inhibit IRES activity (15). Cap-independent translation could be of interest in hematopoietic malignancies since many drugs used for treatment, such as DNA-damaging agents or more recently mTOR inhibitors, shut down cap-dependent translation. By acting to maintain key survival pathways in the face of global translation shutdown, cap-independent translation has the potential to impede many chemotherapeutics’ action.

This review flags some instances in which IRESes are found in genes relevant to blood cancers, but in which their context is not understood. In many cases, these IRESes may be incidental to disease progression and response to treatment, since cap-dependent translation may be the only relevant mechanism. In other cases, particularly where upregulation of both ITAF and its cognate IRES are seen, both may be playing a critical role. Their potential to help or hinder treatment of hematological malignancies should not be overlooked. To underscore this point, we end this review by elaborating on the rare examples where the roles of cap-independent translation are better understood.

## IRESes are Present in Many Genes Relevant to Blood Cancers

Many genes of relevance to blood cancers contain an IRES element, including many involved in the characteristic translocations (see Table 1). Crucially, the contributions of these IRESes to transformation and progression, if any, are not yet known.

**Table 1:** **Genes with the most common translocations observed in blood cancer**.

Translocation	Oncogenic mechanism	Disease (frequency of translocation)	Gene(s) involved	Presence of an IRES in involved genes	IRES retained after translocation
t(8 14)(q24 q32)	Transcriptional activation	BL (80–90%) (16)	*c-Myc*	Yes (18)	Yes
		DLBCL (7–10%) (17)			
t(11 14)(q13 q32)	Transcriptional activation	MCL (80–90%) (17)	*Cyclin D1*	Yes (20)	Yes
		MM (15%) (19)			
t(14 18)(q32 q21)	Transcriptional activation	FL (80–90%) (17)	*BCL2*	Yes (21)	Yes
		DLBCL (12–23%) (17)			
t(3 14)(q27 q32)	Transcriptional activation	DLBCL (20–31%) (17)	*BCL6*	No	NA
t(9 22)(q34 q11)	Fusion protein	CML (100%) (22)	*BCR/ABL1*	No	No
		Adult ALL (30%) (23)			
t(15,17)(q22 q21)	Fusion protein	APL (95%) (24)	*PML/RAR*α	No	NA
t(12 21)(p12 q22)	Fusion protein	Pediatric ALL (25%) (25)	*TEL/AML1*	Yes (AML1) (26)	No

*The presence or absence of IRES elements are indicated in the table*.

*BL, Burkitt lymphoma; DLBCL, diffuse large B-cell lymphoma; MCL, mantle cell lymphoma; MM, multiple myeloma; FL, follicular lymphoma; CML, chronic myeloid leukemia; ALL, acute lymphoid leukemia; APL, acute promyelocytic leukemia; NA, not applicable*.

Many other genetic rearrangements in hematological malignancies (e.g., less common translocations, deletions, duplications, and a wide range of mutations) involve IRES-containing genes. These include *VEGF* or *b-FGF*, which both are mutated in several cancers including CLL (27–30). p53 whose 5′ UTR contains two IRESes (31), is mutated in 5–50% of blood cancers [for review, see Ref. (32)], and mutations or deletions of p53 are particularly common in CLL (33).

## ITAFs may also be Aberrantly Expressed in Blood Cancers

As discussed above, the ITAF proteins which can interact with specific IRESes are critical for cap-independent translation. Several ITAFs show expression changes in blood cancers. One such example is nucleolin which is overexpressed, and its subcellular localization altered, in CLL and myeloid leukemias (34, 35). SP1, whose IRES is targeted by nucleolin (36), is overexpressed and associated with bad prognosis in many cancers, including acute leukemia (37, 38), and it can drive drug resistance in leukemia stem cells (38, 39). In another example, HuR is overexpressed in subtypes of AML, CML, and ALL [reviewed in Ref. (40)]. HuR is an IRES-binding protein and has been reported to inhibit p27^KIP1^ IRES-mediated translation (41). Low expression of p27^KIP1^ is a bad prognosis factor in AML (42). To date, however, the relationship between the oncogenic properties of these two proteins and their cognate IRESes has not been explored.

Fusion proteins generated by translocations can also influence cap-independent translation regulation, e.g., signaling through the tyrosine kinase BCR-ABL1. *BCR-ABL1* is a fusion gene that originates from a reciprocal translocation [t(9;22)(q34;q11)] between chromosomes 9 and 22, to generate an aberrant “Philadelphia” chromosome 22 (Table 1). It is present in >95% of CML cases and ~30% of cases of ALL, and sometimes also in AML. Studies suggest that BCR-ABL1 alone can be sufficient to cause CML [for a review, see Ref. (22)]. Because of this, the development of tyrosine kinase inhibitors, such as imatinib, has improved CML prognosis, although resistance can develop.

The BCR-ABL1 protein controls the transcription of several IRES-containing transcripts, including lymphoid enhancer factor-1 (LEF-1) (43). LEF-1 expression increases with CML progression (44, 45). Translation of full-length LEF-1 is partly controlled by an IRES in its 5′ UTR (43). Several LEF-1 ITAFs have been identified, including eIF4A1 (46), which is itself stimulated by BCR-ABL1 via the mTOR pathway. When both mTOR and eIF4A were inhibited, a reduction in the IRES-driven translation of LEF-1 was observed which correlated with a reduced proliferation in hematopoietic cell lines (46). Direct targeting of eIF4A1 activity, in combination with other chemotherapeutics, may therefore be of use in future treatments of CML in individuals who are resistant to tyrosine kinase inhibitors.

BCR-ABL1 also directly regulates transcription of several ITAFs including La/SSB (47), hnRNPA1 (48), hnRNPE2 (49), and hnRNPK (50). La/SSB has been shown to bind to the IRES of mRNA coding for the chaperone protein BIP (51). This protein is increased in cells expressing BRC/ABL1 fusion protein (52), and a cytosolic isoform of BIP has been described to activate PERK signaling and drive survival in leukemia cells (53). hnRNPA1, hnRNPE2, and hnRNPK have been shown to be important for BCR-ABL1-driven oncogenesis (54). Notari and colleagues also showed that, upon induction by BCR-ABL1, hnRNPK induced *c-Myc* IRES-dependant translation (50). Interestingly, hnRNPA1 has also been shown to associate with *c-Myc* IRES (55, 56). However, the role of the ITAF activity of these proteins in the context of CML has not been studied.

Overexpressed La/SSB, when induced by BCR/ABL1 or by JAK2 mutations, was also shown to bind to a 27 nucleotides sequence in the *MDM2* 5′ UTR and activate translation. Interestingly, *MDM2* 5′ UTR shares 70% identity with the 5′ UTR of *BIP* (47, 57). Regulation of MDM2 expression by La/SSB operates following DNA damage and consequent inhibition of cap-dependent translation, and altogether this would suggest that *MDM2* 5′ UTR does contain an IRES regulated by La/SSB. MDM2 is an ubiquitin ligase that targets p53, leading to its degradation and is overexpressed through various mechanisms in most blood cancer types (58). The potential impact on p53 means that further studies to explore cap-independent translation of MDM2 in hematological malignancies may be of value.

## Multiple Myeloma as a Paradigm for the Importance of Cap-Independent Translation in Blood Cancers

The blood cancer for which we have the clearest evidence for the importance of cap-independent translation is multiple myeloma (MM). MM is a disease caused by clonal expansion of plasmocytes. Normal, mature plasmocytes differentiate from activated B cells and produce large quantities (up to 2000 molecules/s) of antibody, such that immunoglobulins normally occupy ~20% of total plasma protein. This massive protein production means that plasma cells must adapt to great ER stress during their development, sustaining unfolded protein response (UPR) and autophagy without inducing apoptosis (59–61).

The normal lifespan of plasmocytes can vary from a few days to several years. Differentiated plasma cells appear to live longest in specialized niches in the bone marrow (62). It is thought that such niches are limited, generating competition between plasma cells arising at different times (63–65). Overall, the nature of normal plasma cells predisposes them to longevity, resistance to apoptotic effects of ER stress, chemotactic movement to migrate and establish in competition-intense niches, and traits which also aid malignant cell survival. MM usually initiates from a B cell with somatic hypermutation (often of *c-Myc*) and translocations in the immunoglobulin genes (66, 67). While the disease is traditionally described as arising from a transformed differentiated plasma cell, the malignant population may instead derive from a small population of relatively chemoresistant, stem cell-like B cell precursors (68).

Broad spectrum chemotherapeutics, such as Melphalan, are used to treat MM, often in combinations that vary with stage and chemoresistance. Due to the generally greater protein load of MM cells, drugs which target the proteasome (such as bortezomib) are often effective (69). Unusually, thalidomide, a drug with immunomodulatory properties whose mechanism of action is not completely understood, is also increasingly used. This, and similar drugs such as Lenalidomide, appears to have diverse antitumor effects (70, 71). IRESes have the potential to impact upon MM drug response. For example, thalidomide may reduce the proliferative effects of MM cells in part by targeting the IRES of the b-FGF growth factor (72).

**Figure 1:**
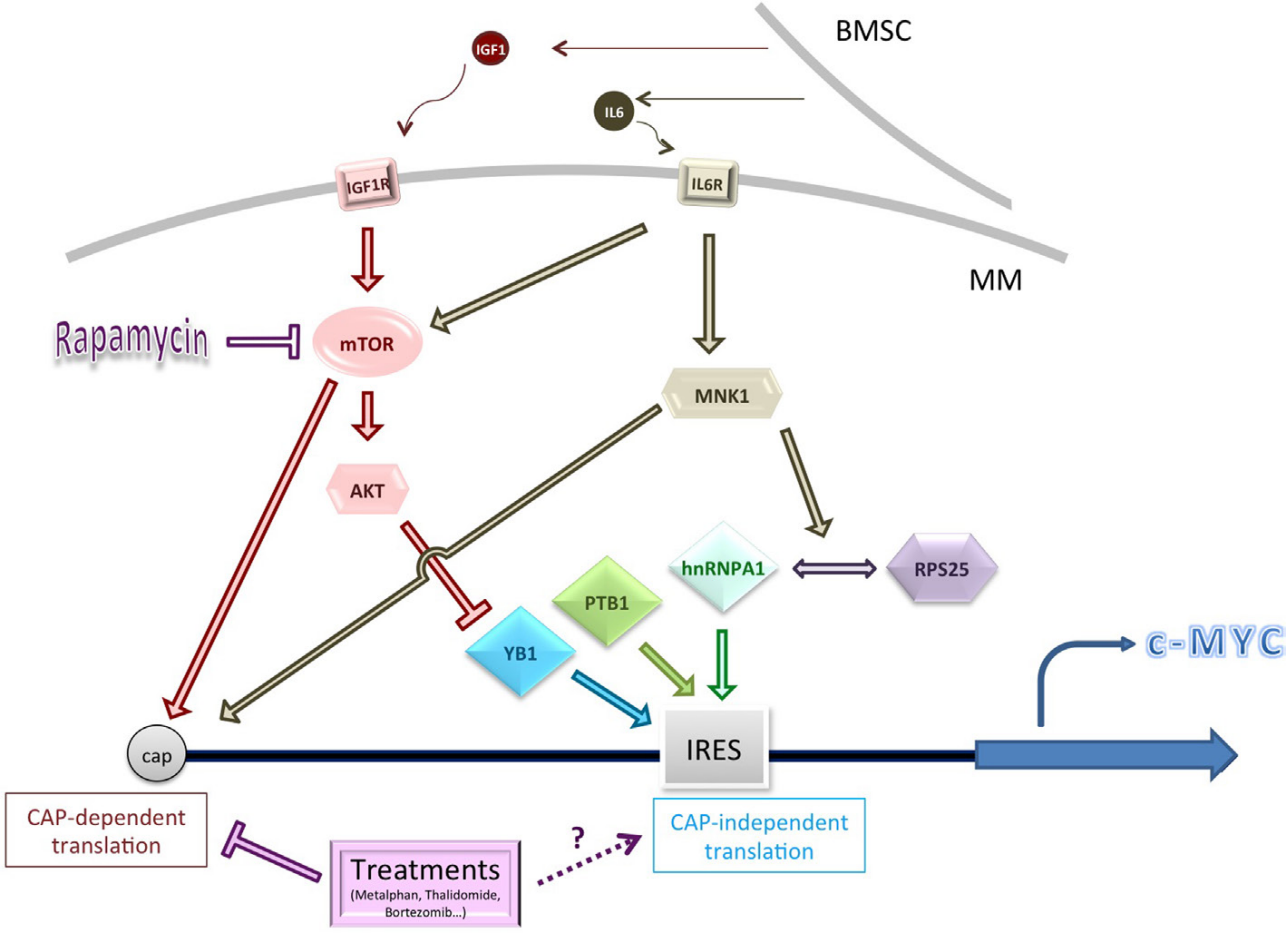
**Schematic summarizing some of the genes and pathways known to influence c-Myc cap-independent translation in multiple myeloma (MM)**. Cytokine activation of key signaling pathways, or pathway dysregulation, may affect the levels or activity of IRES trans-activating factors (ITAFs), including YB1 (conditions of low Akt activity). MNK1-mediated phosphorylation of hnRNPA1 and/or RPS25 increases interaction between them and might improve ribosome loading on the c-Myc IRES (77). Conventional treatments for MM usually inhibit CAP-dependent translation but their impact on CAP-independent translation is unknown. BMSC, bone marrow stromal cell.

The greatest focus of research, however, has been upon c-Myc (summarized in Figure 1). MM cells are thought often to be addicted to c-Myc overexpression and c-Myc levels correlate with disease progression (73–75). c-Myc overexpression is known to have impact on translation in many ways, for example, by acting as a transcriptional activator of ribosomal proteins and translation initiation factors, such as eIF4E [for a review, see Ref. (76)]. Intriguingly, it is also able to modulate mTORC1 activity to enhance 4EBP1 phosphorylation (76).

*c-Myc* contains an IRES in its 5′ UTR (18, 78). A C-T mutation at position 2796 of the IRES affects the secondary structure and correlates with increased c-Myc translation (79). The IRES mutation was found to be overrepresented in the bone marrow of patients with MM (80). Multiple proteins have been identified as Myc family ITAFs (55, 56, 81, 82). Of these, ITAFs PTB1 and YB1 showed a higher affinity for RNA containing the mutated IRES *in vitro*, compared to wild type, and acted synergistically to drive higher expression of a downstream luciferase in a reporter construct. A correlation was also observed between expression of ITAFs PTB1 and YB1 and c-Myc in two MM-derived cell lines carrying the mutation (83).

It should be stressed that elevated YB1 protein levels in MM are likely to have impact widely on its establishment and progression, via both transcriptional and translational effects (84). YB1 effects may also vary with activity of the Ras-ERK and Ras-AKT pathways. High levels of AKT activity are observed in MM (85, 86), and other cancer studies have shown that YB1 is phosphorylated by AKT, rendering it less likely to enhance cap-independent translation (87). In support of this model in MM lines, when mTOR is inhibited by Rapamycin, and AKT activation is maintained, levels of c-Myc and Cyclin D proteins are not maintained by IRES-mediated translation and decline (88). This may be relevant to MM cells in the bone marrow, where malignant cells stimulate the bone marrow stromal cells (BMSCs) to produce the cytokines IL-6 and IGF1, which stimulates the IL6 and IGF1R receptors respectively, leading to activation of AKT (and ERK) signaling (89–91).

In contrast, low AKT activity allows cap-independent translation of c-Myc and Cyclin D to take place (20). It is possible that niche-responsive variation in AKT activity and expression of YB1 (and therefore also c-Myc) allows cells from the same clone, but in different environments, to plastically adopt a quiescent or proliferative phenotype that would result in differential sensitivity to chemotherapeutics (92, 93). A study of the phosphorylation status and relative levels and activity of nuclear and cytoplasmic YB1 and its proteolytically cleaved product within MM samples from different niches would be interesting to explore.

There also appears to be an important role for MNK1 in the activation of the *c-Myc* IRES under chemotherapeutic conditions. MNK1 is best known as a key player in cap-dependent translation; both p38 and ERK1/2 signaling pathways can catalyze its phosphorylation. This activated form can interact with scaffold protein eIF4G and subsequently activate eIF4E phosphorylation (94).

MNK1 can also phosphorylate eIF4B to activate translation [for a review, see Ref. (95)]. Activation of MNK1 via phosphorylation has also been implicated in cap-independent c-Myc translation to facilitate survival during treatment with Rapamycin (and also with common MM chemotherapeutics expected to inhibit cap-dependent translation, such as bortezomib) (77, 88). MNK1 is likely to be phosphorylated within the bone marrow microenvironment, since IL6 stimulates the activity of the p38 MAPK pathway (96).

MNK1 was found to be necessary for the interaction of the hnRNPA1 ITAF and RPS25 with the *c-Myc* IRES (77, 82). Both proteins have previously been established as having roles in cap-independent translation (which for RPS25 appears to outweigh any role in cap-dependent translation) (97). Recent work has identified a compound able to prevent hnRNPA1 binding to the *c-Myc* IRES; the compound was observed to reduce c-Myc expression only following induction of ER stress, when only IRES-dependent mechanisms would be expected to act (77). There is thus an exciting possibility that cap-independent translation inhibitors may be combined with chemotherapeutics that inhibit cap-dependent translation to block a possible survival route for cancer cells.

In a note of caution, the existence of multiple *c-Myc* ITAFs renders it possible that the *c-Myc* IRES could be stimulated *in vivo* even in the presence of an hnRNPA1 inhibitor, since it is currently unknown whether this particular ITAF is essential to c-Myc IRES function under all conditions. Other ITAFs, such as YB1, may be able to act independently of hnRNPA1 as *c-Myc* ITAFs (98). Detailed characterization of the key signaling pathways permitting survival in MM cells across different niches, and after different treatment regimens, would provide clarity on this subject.

## Conclusion

Existing data illustrate that IRES function is relevant to several hematological malignancies and that specific IRESes or ITAFs may present druggable targets in the future. However, much work still needs to be done to clarify the roles of known IRESes in pathology and response to chemotherapeutics, in addition to finding further relevant examples of cap-independent translation. While acknowledging unusual examples such as LEF-1, one would expect that, in the majority of cases, IRES function is likely to be of most relevance when cap-dependent translation is compromised, e.g., following exposure to chemotherapeutic agents and in a hypoxic tumor cell environment. How IRESes mediate survival in such circumstances is likely to lead to a better understanding of which combinations of chemotherapeutics can be used to treat this very disparate and difficult to treat group of diseases. One way to address this question would be to ask if clones enriched for IRES-containing oncogenes arise, and persist, even as a small niche-specific subpopulation, following chemotherapy.

## Author Contributions

All authors contributed to writing.

## Conflict of Interest Statement

The authors declare that the research was conducted in the absence of any commercial or financial relationships that could be construed as a potential conflict of interest.
